# Ginsenoside Rg3 stereoisomers differentially inhibit vascular smooth muscle cell proliferation and migration in diabetic atherosclerosis

**DOI:** 10.1111/jcmm.13601

**Published:** 2018-03-22

**Authors:** Mengqi Guo, Guanlun Guo, Jie Xiao, Xi Sheng, Xinyu Zhang, Yuanyuan Tie, Yuen‐Kit Cheng, Xiaoping Ji

**Affiliations:** ^1^ Key Laboratory of Cardiovascular Remodeling and Function Research Chinese Ministry of Education and Chinese Ministry of Health Department of Cardiology Qilu Hospital of Shandong University Jinan Shandong China; ^2^ Hubei Key Laboratory of Advanced Technology for Automotive Components & Hubei Collaborative Innovation Center for Automotive Components Technology Wuhan University of Technology Wuhan Hubei China; ^3^ Department of Chemistry Faculty of Science Hong Kong Baptist University Hong Kong city Hong Kong

**Keywords:** atherosclerosis, ginsenoside, PPARγ, Rg3, stereoisomer

## Abstract

Ginsenoside 20(R/S)‐Rg3, as a natural peroxisome proliferator‐activated receptor gamma (PPARγ) ligand, has been reported to exhibit differential biological effects. It is of great interest to understand the stereochemical selectivity of 20(R/S)‐Rg3 and explore whether differential PPARγ activation by Rg3 stereoisomers, if it exists, could lead to differential physiological outcome and therapeutic effects in diabetic atherosclerosis. Here, we investigated the binding modes of 20(R/S)‐Rg3 stereoisomers in the PPARγ ligand‐binding domain (PPARγ‐LBD) using molecular modelling and their effects on smooth muscle cell proliferation and migration induced by advanced glycation end products (AGEs). The results revealed that 20(S)‐Rg3 exhibited stronger antiproliferative and antimigratory effects due to stronger PPARγ activation. To validate the in vitro results, we used a mice model with diabetic atherosclerosis and obtained that 20(S)‐Rg3 markedly reduced the plaque size secondary to reducing the proliferation and migration of VSMCs, while the plaques were more stable due to improvements in other plaque compositions. The results shed light on the structural difference between Rg3 stereoisomers that can lead to significant differential physiological outcome, and the (S)‐isomer seems to be the more potent isomer to be developed as a promising drug for diabetic atherosclerosis.

## 1 INTRODUCTION

Ginseng, the root of *Panax ginseng* C. A. Meyer, has been consumed as herbal drug in traditional oriental medicine for preventive and therapeutic purposes for over 2000 years. Ginsenosides, the pharmacological active phytochemicals of ginseng, have been demonstrated to exert beneficial effects on diabetes and cardiovascular diseases (CVDs).^1^, ^2^ Three types of ginsenosides have been classified, protopanaxadiols (eg, Rb1, Rb2, Rh2 and Rg3), protopanaxatriols (eg, Re, Rg1 and Rg2) and oleanolic acid derivatives.^3^ Structurally, ginsenosides contain a hydrophobic triterpenoid skeleton attached with hydrophilic sugar moieties or hydroxyl groups at carbon‐3, carbon‐6 and carbon‐20, mostly existing as enantiomeric pairs. The stereoselectivity of ginsenosides on many biological effects have been reported previously.^4^, ^5^, ^6^, ^7^, ^8^, ^9^

Recent experiments have demonstrated that ginsenoside Rg3, which contains 2 neighbouring hydroxyl groups near and on the chiral centre C‐20, can act as a natural ligand of PPARγ.^10^ Angiogenesis assay found that both Rg3 stereoisomers can induce differential angiogenesis effects via PPARγ, and the PPARγ agonist activity of 20(S)‐Rg3 is 10 times stronger than that of 20(R)‐Rg3.^11^ A fluorescence polarization and total internal reflection fluorescence (FP‐TIRF) binding study also confirmed that only 20(S)‐Rg3 can quantitatively bind to the PPARγ ligand‐binding domain (PPARγ‐LBD).^12^ To further understand the stereochemical selectivity of Rg3 enantiomers, it is timely and of great interest to model the binding modes of 20(R/S)‐Rg3 in the PPARγ‐LBD.

PPARγ is a member of the nuclear receptor superfamily of ligand‐inducible transcription factors and regulates multiple pathways involved in the development of diabetes and CVDs.^13^, ^14^ Recent studies have implied the role of PPARγ in regulating vascular smooth muscle cell (VSMC) proliferation and migration, an essential event in the development of diabetic atherosclerosis.^15^, ^16^ Under diabetic conditions, the accumulation of hyperglycaemia‐induced AGEs and activation of the receptor for AGEs (RAGE) are key factors mediating these events.^17^, ^18^, ^19^ The objective of this study was to investigate stereo‐selective binding of Rg3 enantiomers to PPARγ based on the stereochemical structures and to explore whether differential PPARγ activation by Rg3 stereoisomers could lead to differential effects on AGEs‐stimulated proliferation and migration of VSMCs and diabetic atherosclerosis formation.

## 2 METHODOLOGY

### 2.1 Simulation and calculation

The initial co‐ordinates of the protein were taken from the crystal structure of the PPARγ‐LBD complexed with the full‐agonist LT160 (chain A of PDB entry: 2I4J^20^). Docking of 20(S)‐Rg3 or 20(R)‐Rg3 in the PPARγ ligand‐binding pocket (PPARγ‐LBP) was performed with the program Autodock v4.0.^21^ The Rg3 docked poses were taken as the initial configurations of the ligand in subsequent Molecular Dynamics (MD) simulations. For comparison, the apo‐bound, LT127 (partial PPARγ agonist)‐bound and LT160‐bound simulated systems were considered as references. The binding free energies (Δ*G*_*bind*_) for the complexes were calculated using the Molecular Mechanics/Poisson‐Boltzmann Surface Area (MM/PB‐SA) method.^22^, ^23^ The Essential Dynamics Analysis (EDA) was based on the last 10‐ns equilibrated trajectory for each system. The analysis followed the “Interactive Essential Dynamics” method closely.^24^

### 2.2 Cell culture and treatment

Mouse vascular smooth muscle cells (MOVAS cells, ATCC) were seeded into 6‐well plates in routine Dulbecco's modified Eagle's medium with 10% foetal bovine serum for 24 hours, and then the culture medium was changed to 0.1% serum medium. VSMCs were pre‐treated with 20(R/S)‐Rg3 (25 μmol/L, dissolved in 0.1% DMSO; Felton, China) in the presence or absence of GW9662 (3 μmol/L; Selleck, USA) for 1 hour, and then stimulated with AGEs (100 μg/mL; Biovision, USA) for 48 hours. An equal volume of DMSO was added to the controls. For all data shown, each individual experiment represents an independent preparation of VSMCs.

### 2.3 PPARγ reporter gene assay

PPARγ reporter gene assay was performed in 293T cells transfected with reporter plasmid peroxisome proliferator‐activated receptor response element (PPRE)X3‐TK‐luc, expression plasmids pSG5‐PPARγ and pSG5‐RXRα. After 6 hours of transfection, cells were washed once with Opti‐MEM, supplemented by different concentrations of 20(S)‐Rg3 or 20(R)‐Rg3, further incubated with AGEs for 24 hours. Cells were lysed by passive reporter lysis buffer; luciferase activity was measured using Luciferase Assay System (Promega, USA) with microplate reader (BioTek, USA). Luciferase activity was normalized by the amount of protein in the cell lysate.

### 2.4 Cell proliferation, migration and cell cycle assay

Proliferation was analysed using the Cell Counting Kit‐8 (CCK‐8; Beyotime, China) and Cell‐Light™ EdU assay (RiboBio, China) according to the manufacturer's directions. Cell cycle analysis was performed by flow cytometry. VSMC migration assays were performed in Transwell chambers (8 μm pore size; Corning Inc., USA).

### 2.5 Western blot and gelatin zymography

Proteins were separated by 10% sodium dodecyl sulphate‐polyacrylamide gel electrophoresis (SDS‐PAGE), transferred to polyvinylidene difluoride membranes, probed with matrix metalloproteinase 2 (MMP2) (1:1000; Abcam, ab37150), matrix metalloproteinase 9 (MMP9) (1:1000; Abcam, ab38898), cyclin D1 (1:10 000; Abcam, ab134175), cyclin E (1:1000; Cell Signalling, 4129P), proliferating cell nuclear antigen (PCNA) (1:200; Bioss, bs‐2007R), β‐actin (1:1000; 2SGB‐BIO, TA‐09) and phosphoric acid dehydrogenase (GADPH) (1:1000; Cell Signalling, 2118P) overnight at 4°C, and developed with chemiluminescence. When necessary, the blots were stripped and reprobed. For gelatin zymography, cell culture supernatants were electrophoresed in a 8% polyacrylamide gel containing 1 mg/mL gelatin. Lytic bands of gelatin digestion represented MMP2 (72‐kD) and MMP9 (92‐kD) activity.

### 2.6 Animal model

All experimental procedures were performed in accordance with guidelines for Institutional Animal Care and were approved by the Animal Ethics Committee of Shandong University. Eight‐week‐old male ApoE−/− mice (n = 60) were randomly divided into the following 6 groups (n = 10 per group): non‐diabetic control group, DM (diabetes mellitus) + placebo group, DM + 20(R)‐Rg3 group, DM + 20(R)‐Rg3 + GW9662 group, DM + 20(S)‐Rg3 group and DM + 20(S)‐Rg3 + GW9662 group. Diabetes was initiated by the administration of 5 daily intraperitoneal injections of 50 mg/kg streptozotocin (STZ) in citrate buffer (0.05 mol/L; pH 4.5). Mice with blood glucose levels of >300 mg/dL at 2 weeks after the initial STZ administration were considered diabetic and were included in the DM cohorts. Mice received a normal chow for the remaining 10 weeks. During the 6th‐10th weeks, mice were given Rg3 at a dose of 10 mg/kg i.p. once 2 days,^25^ with oral gavage of GW9662 at 3 mg/kg per day.^26^

### 2.7 Biochemical measurements

Serum lipid profiles, including total cholesterol, triglyceride levels and glucose concentration, were measured by enzymatic assay with the use of an automatic biochemical analyser (Roche Cobas Integra 800, Switzerland).

### 2.8 Histology and immunohistochemistry

To assess overall burden and distribution of atherosclerosis, en face lesion staining with Oil Red O was performed as previously described.^27^ The remaining staining involved cross sections of the aortic roots (predilection site of atherosclerosis). The sections were stained with haematoxylin and eosin (H&E) following a standard protocol of our laboratory. Haematoxylin was applied for 4 minutes followed by a 20‐second differentiation in ammonia, after which eosin was applied for 20 seconds. The content of lipids and collagen of aortic plaques was detected by Oil Red O staining and Picrosirius Red staining, respectively.^28^

The immunohistochemical staining was performed as previously described.^27^ Sections were incubated with the primary antibodies against α‐SMA (1:200; Abcam, ab5694), monocyte/macrophage antigen [MOMA‐2] (1:200; MCA519G; AbD, UK), MMP2 (1:250; NovusBio, NB200‐193) and MMP9 (1:100; Abcam, ab38898). For immunofluorescence, frozen sections were labelled with primary antibodies against α‐SMA (1:500; NovusBio, NBP2‐22120) and PCNA (1:100; CST, 13110S) simultaneously overnight, followed by incubation with corresponding fluorophore‐conjugated secondary antibodies. The stained sections were mounted with DAPI‐containing VectaShield mounting medium (Vector) and then viewed using an Olympus BX53 fluorescence microscope. The results were analysed using the Image‐Pro Plus 6.0 software.

### 2.9 Statistical analysis

All experiments were repeated at least 3 times, and data were presented as the mean ± SEM. Statistical analysis was carried out using ANOVA followed by Tukey's post hoc test (GraphPad Software, USA). *P* < .05 was considered significant.

## 3 RESULTS

### 3.1 Characteristics of the binding modes of 20(R/S)‐Rg3 stereoisomers

**Figure 1:**
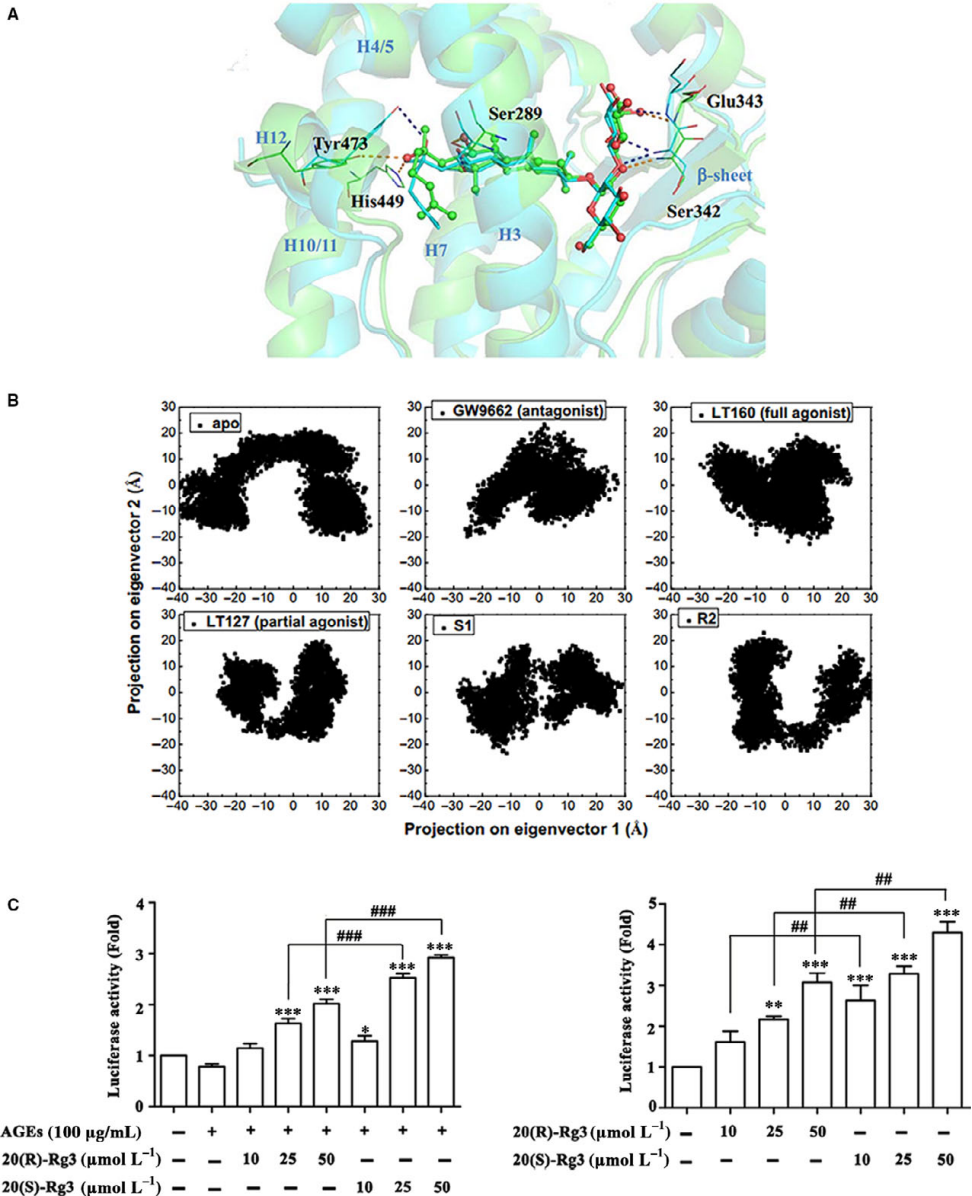
Simulation and calculation of the binding modes of 20(R/S)‐Rg3 in models R2 and S1. A, Superposition of 20(R/S)‐Rg3 in models R2 and S1 after simulation. The LBD in model S1 is shown as a green ribbon and that in model R2 is shown as a blue ribbon. 20(S)‐Rg3 in model S1 is shown as green ball‐and stick models, and 20(R)‐Rg3 in model R2 is shown as blue sticks. Tyr473, Ser342, Glu343 and His449 in model S1 are shown as green wires and those in model R2 are shown as blue wires. The hydrogen bonds in model S1 are shown as orange dotted lines, and those in model R2 are shown as black dotted lines. B, EDA projections onto the first 2 eigenvectors of models S1 and R2 involved in those of the apo‐bound, GW9662‐bound, LT160‐bound and LT127‐bound systems. The calculation was based on the last 10‐ns equilibrated trajectories. C, D, PPARγ transcriptional activity in transfected 293T cells with and without AGEs incubation (n = 3). Values are presented as the mean ± SEM. * *P* < .05, ** *P* < .01, *** *P* < .001 vs control; ##*P* < .01, ###*P* < .001 vs the 20(R)‐Rg3 groups

The initial reasonable poses of 20(R/S)‐Rg3 enantiomers in the LBP were obtained from computational docking via regarding the orientation of the full‐agonist LT160 as template. Totally 6 candidates (S1, S2, S3 for 20(S)‐Rg3 and R1, R2, R3 for 20(R)‐Rg3) were submitted to MD simulations (Table S1). The average charge‐clamp distance and MM/PB‐SA estimated binding energy (Table S2), as well as the root‐mean‐squared fluctuation (RMSF) for each system (Figure S1), all indicate that models S1 and R2 are the most probable binding modes of 20(R/S)‐Rg3 enantiomers in PPARγ. The orientations of the enantiomers in the 2 models were superimposed in Figure 1A. EDA was then employed to differentiate these 2 models, and the backbone atom traces of the models are compiled in Table 1. The smaller backbone atom trace of model S1 implies that 20(S)‐Rg3 stabilizes PPARγ more than 20(R)‐Rg3. The EDA projections of the 2 models are both split into 2 separated regions with respect to the first eigenvector and different to those of full agonist and antagonist. While the projection shape of model S1 seems more like that of the partial agonist LT127‐bound system (Figure 1B). Hence, both of 20(R/S)‐Rg3 enantiomers can act as PPARγ partial agonists in principle, but the binding of 20(S)‐Rg3 in PPARγ is more probable.

**Table 1:** Backbone atom trace of S1 and R2 models

Model	Backbone atom trace (A^2^)
apo	676
PPARγ‐GW9662	241
PPARγ‐GW9662	284
PPARγ‐GW9662	263
S1	356
R2	375

### 3.2 Rg3 stereoisomers differentially activate PPARγ

To further compare the agonist activity of 20(R)‐Rg3 and 20(S)‐Rg3 at the cell level, PPARγ luciferase reporter gene assay was performed. Rg3 treatment, especially 20(S)‐Rg3, significantly increased luciferase activity in a dose‐dependent manner (10‐50 μmol/L). The PPARγ transcriptional activity as activated by 20(S)‐Rg3 was more potent than 20(R)‐Rg3 with and without AGEs (100 μg/mL) stimulation (Figure 1C,D). This suggests that the structural difference between 20(S)‐Rg3 and 20(R)‐Rg3 is critical for the differential activation of PPARγ.

### 3.3 Differential effects of Rg3 stereoisomers on AGEs‐induced VSMC proliferation

**Figure 2:**
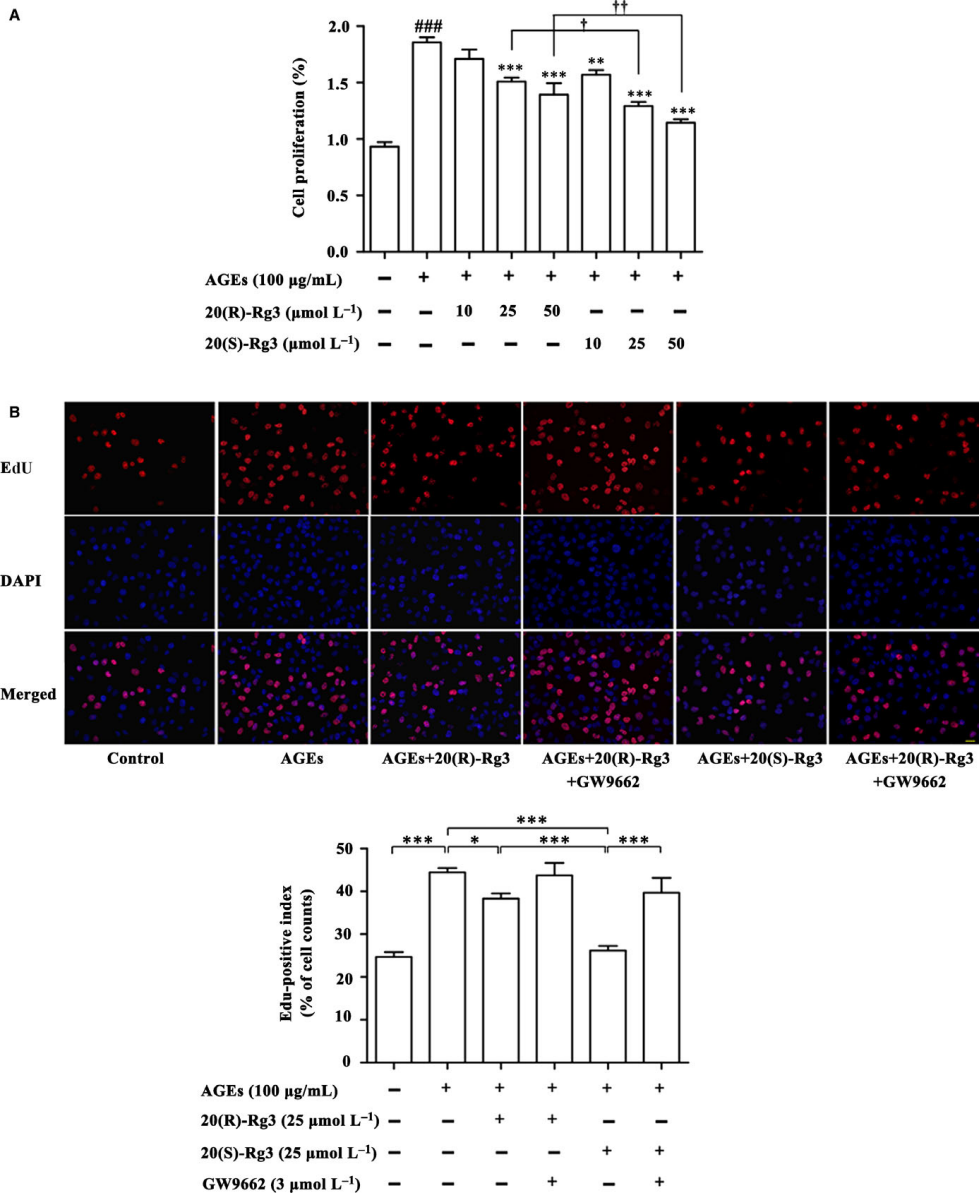
Differential effects of the 20(R/S)‐Rg3 stereoisomers on VSMC proliferation. A, Cell Counting Kit‐8 analysis of the cell viability of VSMCs (n = 5). ###*P* < .001 vs control; * *P* < .05, ** *P* < .01, *** *P* < .001 vs the AGEs group; †*P* < .05, ††*P* < .01 vs the 20(R)‐Rg3 groups. B, Fluorescence microscopy of EdU staining. Nuclei were counterstained with DAPI (blue); red staining indicates cells undergoing proliferation. The EdU‐positive index was expressed as a percentage (positive/total cell number) (n = 5). Scale bar: 10 μm. * *P* < .05, ** *P* < .01, *** *P* < .001. All quantitative data are means ± SEM

The CCK‐8 assay showed that Rg3 inhibited AGEs‐induced proliferation of VSMCs in a concentration‐dependent manner (10‐50 μmol/L), and the effects of 20(S)‐Rg3 were more potent than 20(R)‐Rg3 (Figure 2A). In EdU incorporation assay, 20(R)‐Rg3 (25 μmol/L) and 20(S)‐Rg3 (25 μmol/L) reduced the stimulated DNA synthesis by 12.62% and 37.80%, respectively. Administration of the PPARγ antagonist GW9662 (3 μmol/L) largely reversed these effects, indicating that differential PPARγ activation is responsible for the differential antiproliferative activity of 20(R/S)‐Rg3 (Figure 2B). Treatment of VSMCs with Rg3 at doses of 10, 25 and 50 μmol/L for 48 hours did not show significant cytotoxicity to non‐stimulated VSMCs [14.2% reduction in cell viability at 50 μmol/L for 20(R)‐Rg3, and 15.8% and 16% reductions at 25 and 50 μmol/L for 20(S)‐Rg3, *P* > .05, respectively (Figure S2)], implying that the antiproliferative effect of Rg3 on VSMCs did not result from cytotoxicity.

### 3.4 Effects of Rg3 stereoisomers on AGEs‐induced cell cycle progression in VSMCs

**Figure 3:**
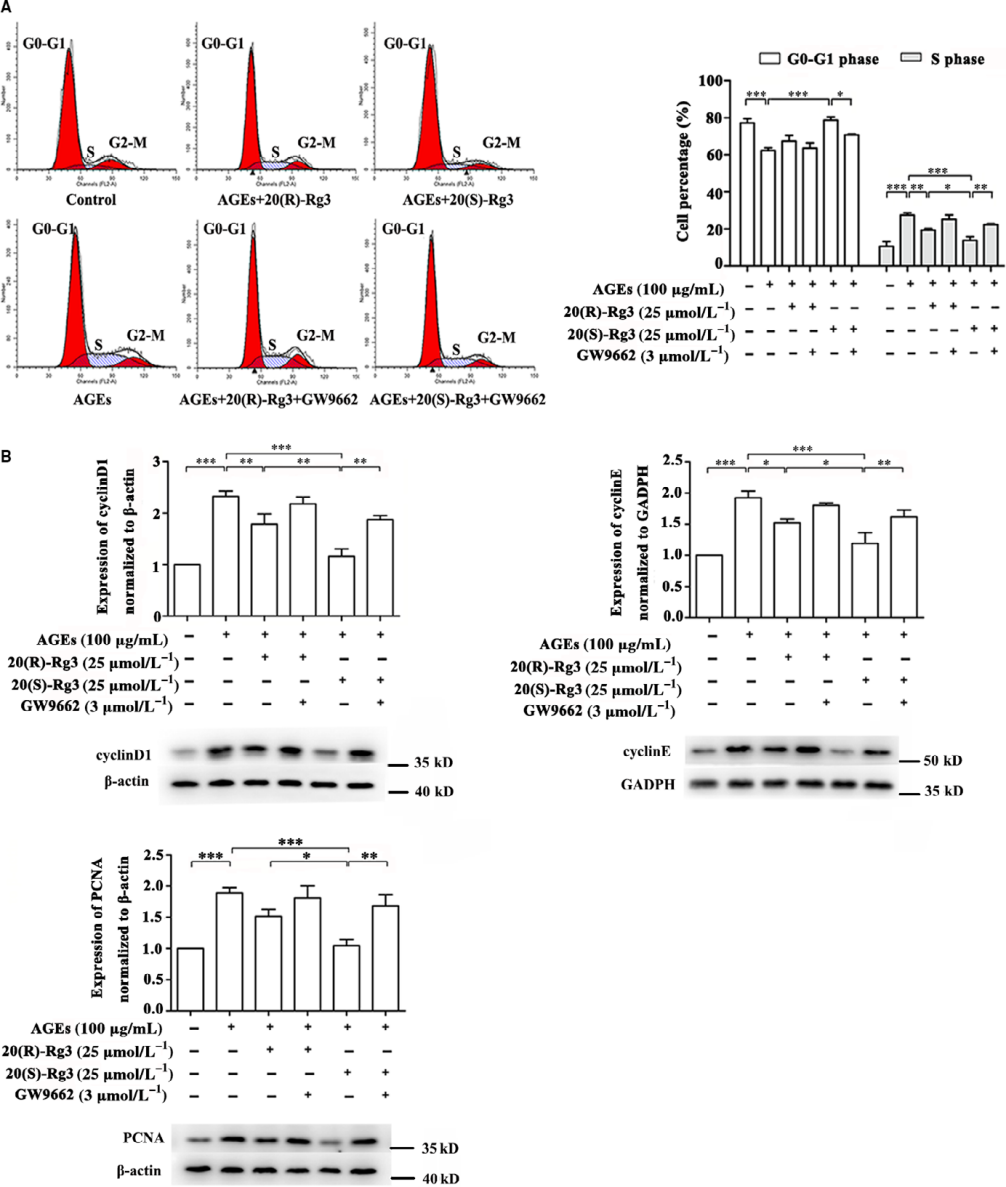
Effects of the 20(R/S)‐Rg3 stereoisomers on cell cycle progression and cell cycle related‐protein expression in AGE‐stimulated VSMCs. A, Flow cytometry analysis of the cell cycle. The relative percentage of cells in different cell cycle phases are reported, while the percentage of apoptotic events was ignored (n = 3). B, Western blot analysis of cyclin D1, cyclin E and PCNA protein expression. The bands are quantified by densitometric analysis. Protein expression of cyclin D1 and PCNA was normalized to β‐actin, and expression of cyclin E was normalized to GADPH (n = 5, 4, and 5 for cyclin D1, cyclin E and PCNA, respectively). The results are expressed as the mean values ± SEM. * *P* < .05, ** *P* < .01, *** *P* < .001

We analysed the effect of the Rg3 stereoisomers on cell cycle progression using flow cytometry analysis. 20(R/S)‐Rg3 both significantly retarded cell cycle progression at G1 phase, and the number of cells in the S phase decreased. 20(S)‐Rg3 treatment was a lot more potent than 20(R)‐Rg3, the effects of which could be abolished by the PPARγ antagonist GW9662 (Figure 3A). To examine the underlying molecular pathways responsible for G1 arrest, we next assessed levels of G1/S‐checkpoint proteins.^29^ 20(S)‐Rg3 significantly inhibited the increased expression of cyclin E and cyclin D1 in AGEs‐stimulated VSMCs, while the effect of 20(R)‐Rg3 was more moderate. Furthermore, the expression of PCNA, which regulates programmes governing the G1/S transition,^30^ was reduced to nearly non‐stimulated level by only 20(S)‐Rg3. Treatment with GW9662 mostly blocked these effects (Figure 3B). These results clearly demonstrate that ginsenoside Rg3, via PPARγ activation, may be targeting some signalling transduction pathways evoked in the G1 to S interphase. Compared with 20(R)‐Rg3, 20(S)‐Rg3 exhibits more potent effects as more effective PPARγ activation.

### 3.5 Differential effects of Rg3 stereoisomers on AGEs‐induced VSMC migration

**Figure 4:**
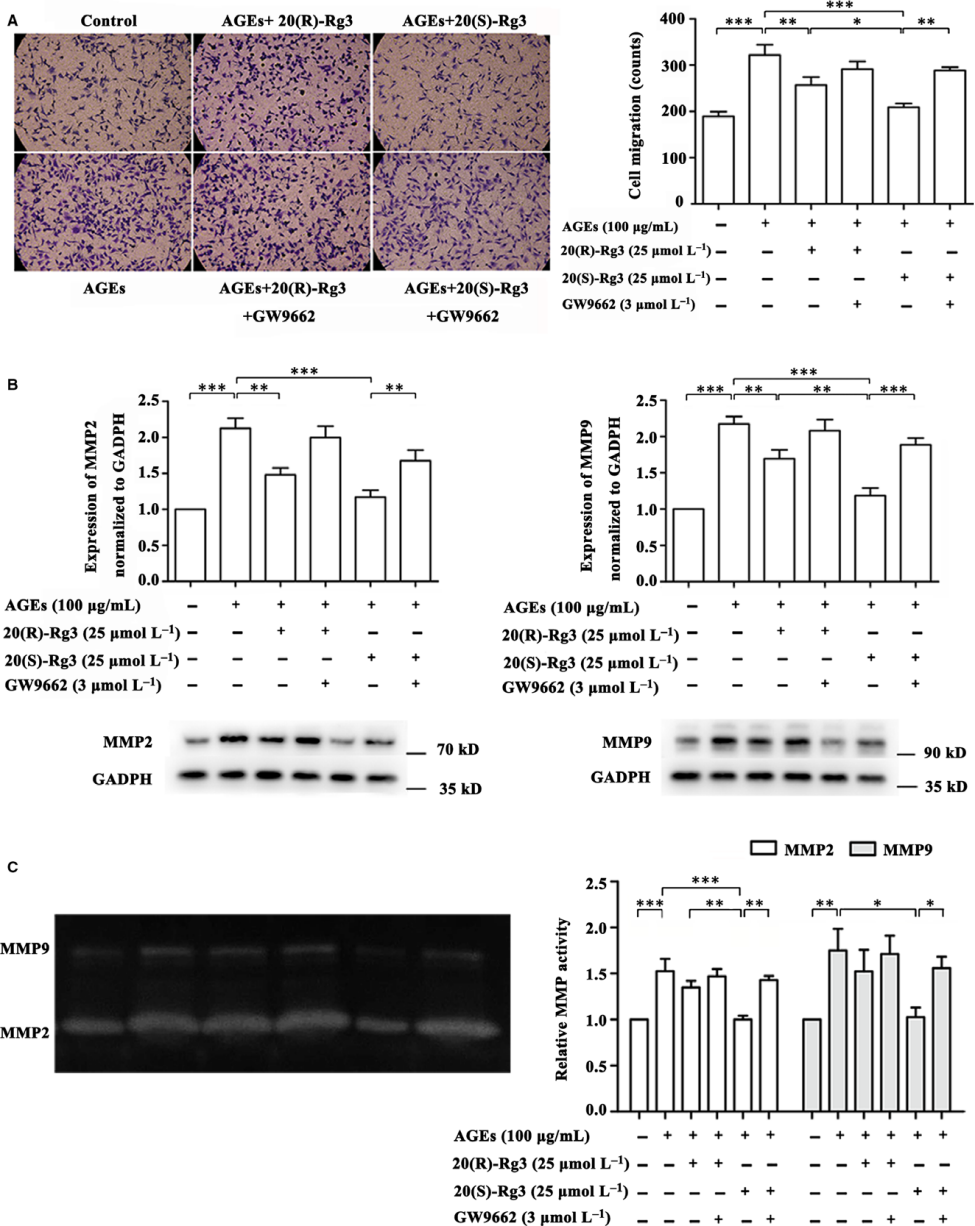
Differential effects of the 20(R/S)‐Rg3 stereoisomers on the migration of AGE‐treated VSMCs. A, Transwell migration assay. Percentage of migrated cells relative to the control are quantified (n = 5). B, Western blot analysis of MMP2 and MMP9 protein expression after 48 h of stimulation. The bands are quantified by densitometric analysis, and protein expression was normalized to GADPH (n = 4 and 5 for MMP2 and MMP9, respectively). C, Gelatin zymography analysis of MMP2 and MMP9 activity in the medium after 48 h of stimulation. Relative activity was quantified by densitometric analysis (n = 3; respectively). The values shown represent means ± SEM of independent assays. * *P* < .05, ** *P* < .01, *** *P* < .001

Transwell migration assays were used to evaluate the effects of 20(R/S)‐Rg3 on the migration of VSMCs. 20(R)‐Rg3 and 20(S)‐Rg3 suppressed the stimulated cell migration by 20.25% and 34.89%, respectively, both of which could be reversed by GW9662 treatment (Figure 4A). In addition, the effects of Rg3 stereoisomers were examined on the MMP‐2/‐9 expression and activity. We observed that 20(S)‐Rg3 markedly attenuated AGEs‐induced increase in MMP2 and MMP9 protein expression, while the effect of 20(R)‐Rg3 was more moderate (Figure 4B). These results are in accordance with the altered MMP activity in gelatin zymography (Figure 4C).

### 3.6 Biochemical parameters after Rg3 treatment in vivo

The serum levels of total cholesterol, triglyceride and fasting glucose of each group are depicted in Table S3. Total cholesterol and triglycerides levels did not differ among the diabetic apoE‐/‐ groups. However, there was a significant reduction in fasting glucose levels in the 20(S)‐Rg3‐treated group compared to the placebo‐treated diabetic group (20.35 ± 2.06 mmol/L vs 24.65 ± 0.82 mmol/L, *P* < .05). In addition, treatment with 20(R/S)‐Rg3 was well tolerated and did not impair the apparent health or survival of the mice.

### 3.7 Effects of Rg3 stereoisomers on atherosclerotic plaque size and frequency of intraplaque VSMCs

**Figure 5:**
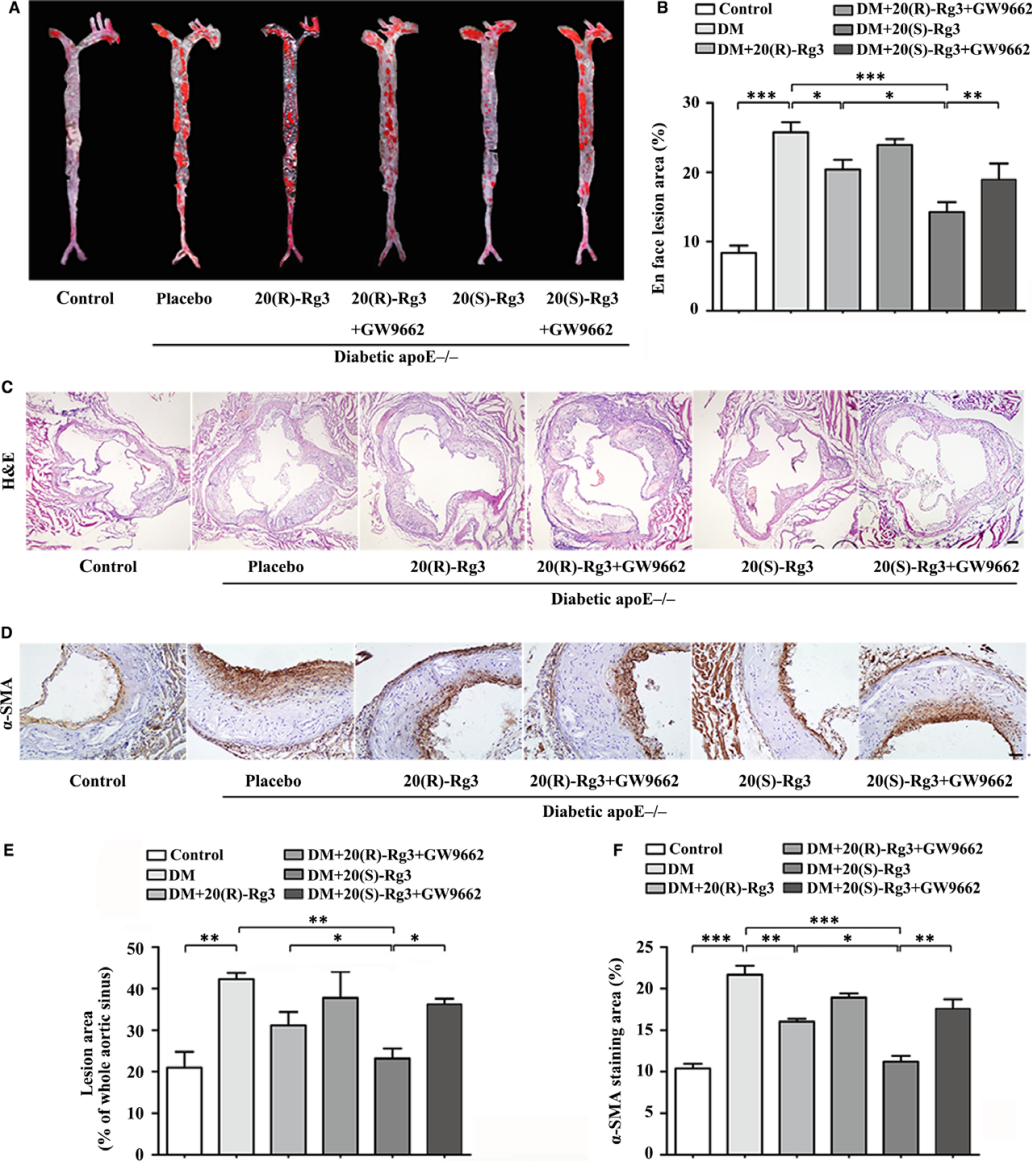
Effects of the 20(R/S)‐Rg3 stereoisomers on early atherogenesis in diabetic mice. A,B, En face analysis of aortas. Atherosclerotic lesions were identified by Oil Red O staining (n = 3). The ratio of the atherosclerotic lesion area to the total vessel area is quantified, indicating level of atherogenesis. C, E, Haematoxylin and eosin staining of aortic sinus cryosections; the ratio of total atherosclerotic lesion area to aorta lumen area are quantified (n = 3). Scale bar: 100 μm. D, F, Representative immunohistochemical α‐SMA staining and quantification of the plaque smooth muscle cell content (n = 3). Scale bar: 50 μm. The data are expressed as the mean ± SEM * *P* < .05, ** *P* < .01, *** *P* < .001

The relative en face lesion area of the entire aorta (Figure 5A,B) and the cross‐sectional plaque area of the aortic sinus (Figure 5C,E) were significantly reduced in the Rg3 treatment groups compared with the placebo‐treated group and these effects were more significant in the 20(S)‐Rg3 group than in the 20(R)‐Rg3 group. We next evaluated the frequency of intraplaque VSMCs, which could partly determine the plaque size. Immunohistochemical analysis revealed a significant increase in the α‐SMA staining within plaques of diabetic mice relative to the non‐diabetic group. 20(S)‐Rg3 treatment markedly decreased the intraplaque α‐SMA intensity, more potent than 20(R)‐Rg3 treatment, while GW9662 co‐administration blocked this effect (Figure 5D,F). These results indicate that 20(S)‐Rg3 may more significantly reduce atherosclerotic plaque size and VSMC frequency due to more potent PPARγ activation. To explore whether reduced smooth muscle in 20(S)‐Rg3 group means a less stable plaque, we then assessed the plaque compositions. To our great relief, besides reducing VSMCs, 20(S)‐Rg3 significantly increased the intraplaque content of collagen and decreased that of lipids and macrophages, and the vulnerability index calculated as (lipid deposit% + macrophages%)/(collagen fibres % + SMCs%) was actually decreased^31^ (Figure S3).

### 3.8 Effects of Rg3 stereoisomers on the proliferation and migration of VSMCs within atherosclerotic plaques

**Figure 6:**
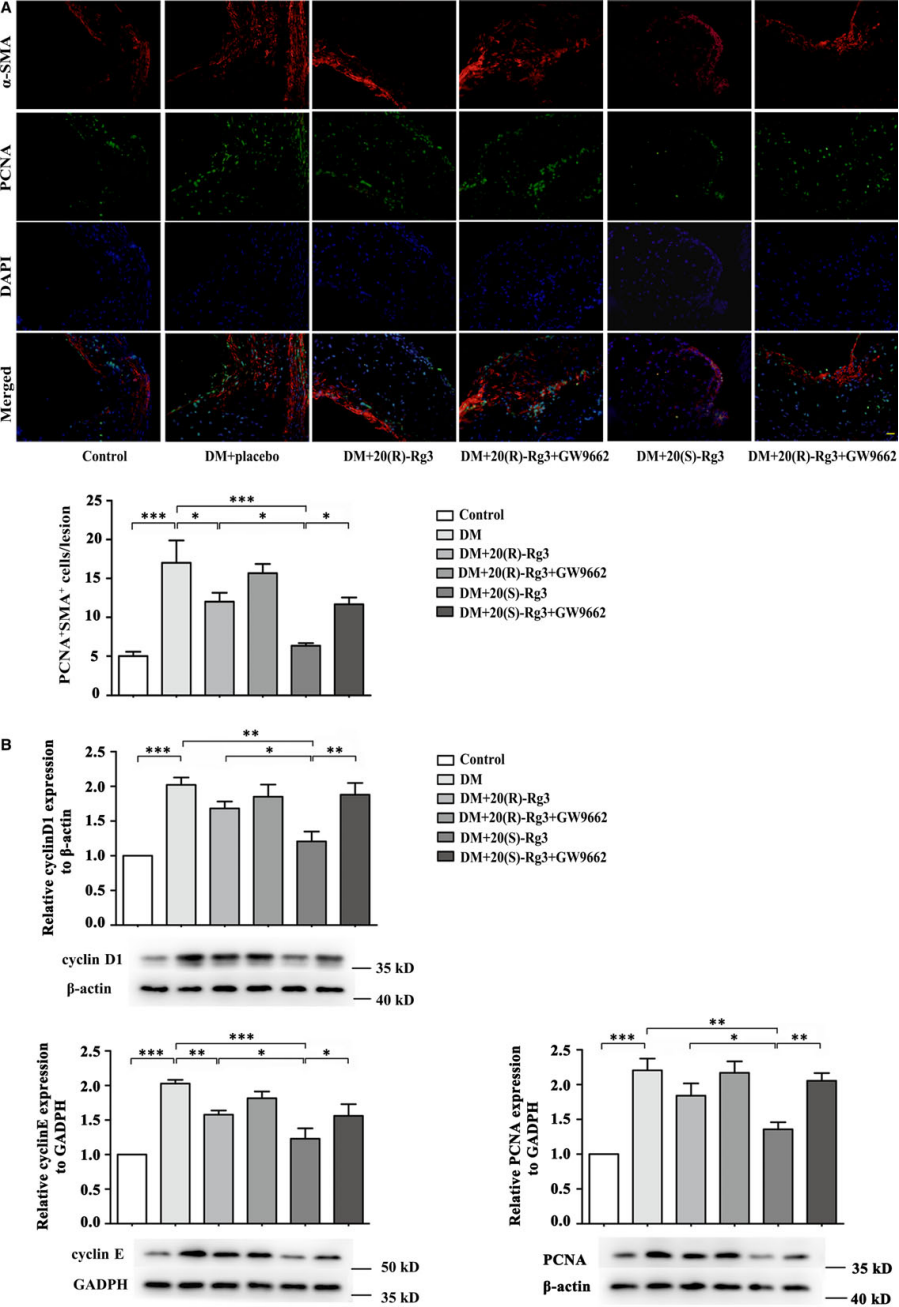
Differential effects of the 20(R/S)‐Rg3 stereoisomers on proliferation of VSMCs within plaques. A, Co‐immunofluorescence staining of the aortic root for VSMCs (anti‐α‐SMA antibody, red) and proliferation marker PCNA (green) and a bar graph summarizing the results (n = 3). Scale bar: 20 μm. B, Western blot analysis of cyclin D1, cyclin E and PCNA protein expression within plaques. The bands are quantified by densitometric analysis. Protein expression of cyclin D1 and PCNA was normalized to β‐actin, and expression of cyclin E was normalized to GADPH (n = 3, 4, and 4 for cyclin D1, cyclin E and PCNA, respectively). The results are expressed as the mean values ± SEM. * *P* < .05, ** *P* < .01, *** *P* < .001

**Figure 7:**
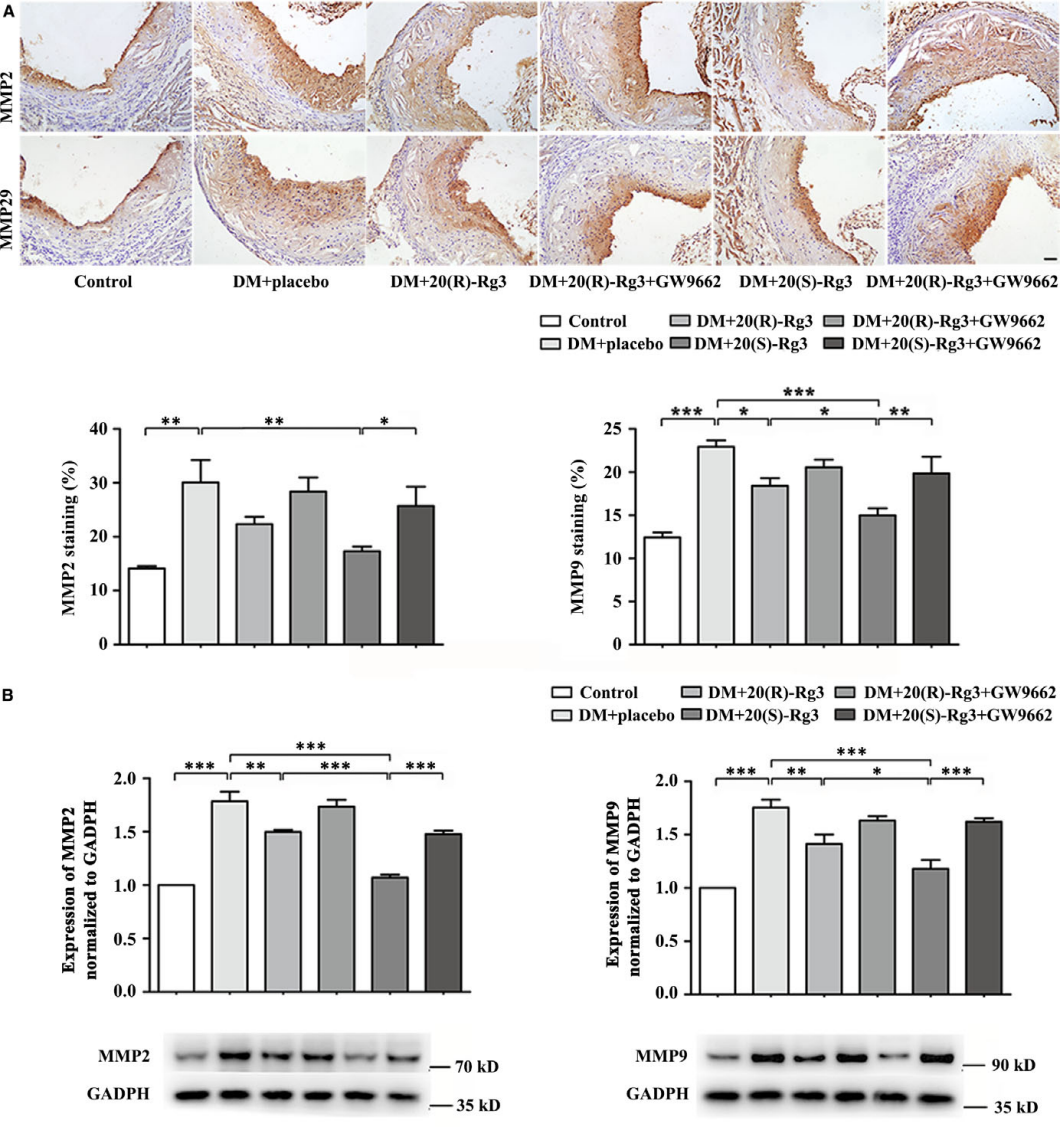
Differential effects of the 20(R/S)‐Rg3 stereoisomers on MMP expression within plaques. A, Representative immunohistochemical MMP2 and MMP9 staining and quantification analysis (n = 3, respectively). Scale bar: 50 μm. B, Western blot analysis of MMP2 and MMP9 protein expression within plaques. The bands are quantified by densitometric analysis, and protein expression was normalized to GADPH (n = 3, respectively). The results are expressed as the mean values ± SEM. * *P* < .05, ** *P* < .01, *** *P* < .001

Co‐immunofluorescence staining indicated a marked reduction in the frequency of proliferating VSMCs in the 20(S)‐Rg3‐treated group compared to that in the placebo‐treated diabetic mice (Figure 6A). Immunohistochemical analysis revealed that 20(S)‐Rg3 markedly decreased the MMP2 and MMP9 intensity within atherosclerotic lesions, more potent than 20(R)‐Rg3 treatment (Figure 7A). In addition, Rg3 treatment, especially 20(S)‐Rg3, significantly reduced the protein levels of these markers as compared to the placebo‐treated group (Figures 6B and 7B). For all above, GW9662 co‐administration mostly reversed the effects of Rg3, indicating that Rg3 stereoisomers may differentially regulate the proliferation and migration of VSMCs via differential PPARγ activation.

## 4 DISCUSSION

The importance of drug stereoselectivity is gaining greater attention in recent years,^32^ and differential pharmacological effects have been reported between ginsenoside Rg3 stereoisomers.^9^, ^11^, ^12^ It is because although the enantiomers of Rg3 possess the same functional group at C20, the difference in three‐dimensional structure may affect the binding affinity to the receptor binding site. Previous computational modelling demonstrated that the hydroxyl group at the C20 chiral centre of 20(S)‐Rg3 could interact with Tyr473 through a hydrogen bond, similar to that of rosiglitazone,^33^, ^34^ while the sterically strained binding pocket prevented the optimal interaction of 20(R)‐Rg3 with Tyr473.^11^ In this report, we further performed MD simulation and EDA to investigate the binding modes of 20(R/S)‐Rg3 stereoisomers in PPARγ‐LBD. Here, we predicted that both of the 20(R/S)‐Rg3 stereoisomers might act as PPARγ partial agonists, but the binding of 20(S)‐Rg3 to PPARγ is more probable. This speculation was further confirmed by the results of the reporter gene assay, indicating that the differential binding modes of Rg3 stereoisomers in the PPARγ‐LBD contribute to differential PPARγ activation at the cell level.

Ginsenosides such as Rb1, Rg1 and compound K have been reported to inhibit VSMC proliferation and neointimal hyperplasia^35^, ^36^, ^37^, ^38^; however, there has been no related research on a diabetic atherosclerosis model. This report reveals that both 20(R/S)‐Rg3 stereoisomers could inhibit AGEs‐induced VSMC proliferation, but the effect of 20(S)‐Rg3 was more potent, with a more remarkable G1 arrest and more significant down‐regulation of G1/S transition regulatory proteins. Increasing evidence suggested that natural and pharmacological PPARγ ligands could induce rapid non‐transcriptional effects in different cell types due to the extranuclear trafficking of PPARγ, and there maybe direct interaction of PPARγ with ERK or its upstream kinases in the signal pathways of cell cycle regulation.^39^, ^40^, ^41^ On the other hand, PPARγ agonists may inhibit cell growth via blockade of RAGE signalling under AGEs stimulation.^15^, ^42^ The downstream signal transduction mechanisms of PPARγ activation by Rg3 stereoisomers remains to be explored.

Our in vivo results imply that Rg3, especially 20(S)‐Rg3, could reduce the plaque size during de novo diabetic atherogenesis, secondary to reducing the proliferation and migration of VSMCs. There is a concern that whether reduced smooth muscle after 20(S)‐Rg3 treatment may lead to a much less stable plaque and one that is more likely to rupture and cause further damage and possibly death, we further assessed the plaque compositions. To our great relief, 20(S)‐Rg3 significantly improved other compositions in the plaque (increased the intraplaque content of collagen and decreased that of lipids and macrophages), and the plaque stability was actually increased. It has been reported that PPARγ agonist could block classical activation of macrophages and attenuates inflammation in diabetic atherosclerosis, thus may promote a more favourable plaque morphology.^43^, ^44^ In addition, atherosclerosis is also affected by lipid profiles and glucose metabolism.^45^, ^46^ There was a trend towards a decrease in blood glucose levels in the 20(S)‐Rg3–treated diabetic mice, which might also contribute to its anti‐atherosclerotic effects.^47^

In summary, this study provides new insights into the differential pharmacological effects of the 20(R/S)‐Rg3 stereoisomers, in which the differential binding modes in the PPARγ‐LBD account for differential PPARγ activation, and stronger PPARγ activation elicits more effective downstream activity, leading to more significant suppression of AGEs‐induced VMSC proliferation and diabetic atherosclerosis formation. The results imply that ginsenoside 20(S)‐Rg3 may have more potentials to be developed as a promising drug for diabetes and atherosclerosis.

## CONFLICT OF INTEREST

None.

## AUTHOR CONTRIBUTION

JXP and GGL conceived and designed the experiments.GMQ, CYK, XJ, SX, ZXY, and TYY performed the experiments.GMQ, CYK and XJ researched data and contributed to discussion. SX and ZXY analysed the data.GMQ and TYY contributed to reagents/materials/analysis tools.GMQ and GGL wrote the manuscript.JXP and GGL reviewed and edited the manuscript.
