# Supplementary figures and images for: Ginsenoside Rg3 stereoisomers differentially inhibit vascular smooth muscle cell proliferation and migration in diabetic atherosclerosis
Source: J Cell Mol Med. 2018 Mar 22;22(6):3202–14. doi: 10.1111/jcmm.13601 (PMC5980205; doi:10.1111/jcmm.13601)

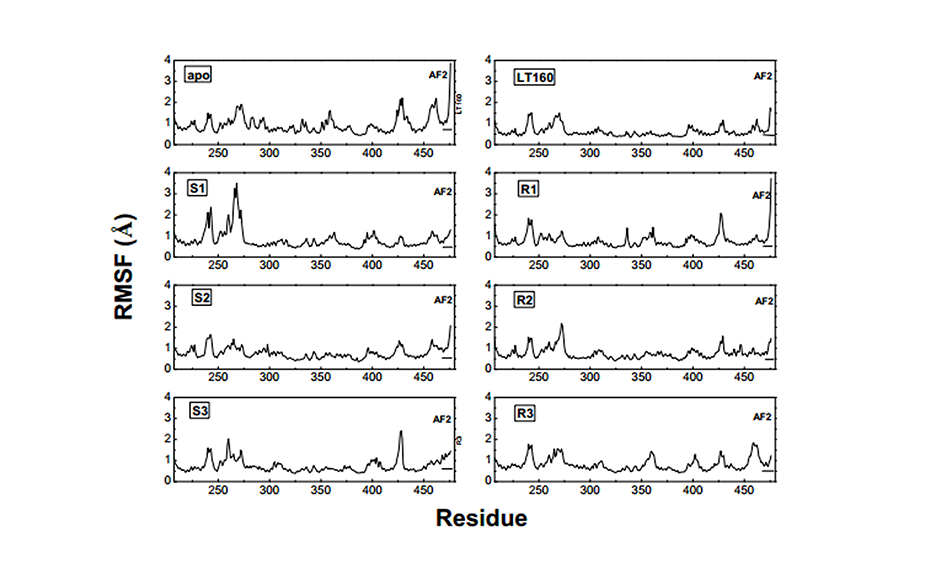

Supplement: Supplementary file 1 [file JCMM-22-3202-s001.tif]

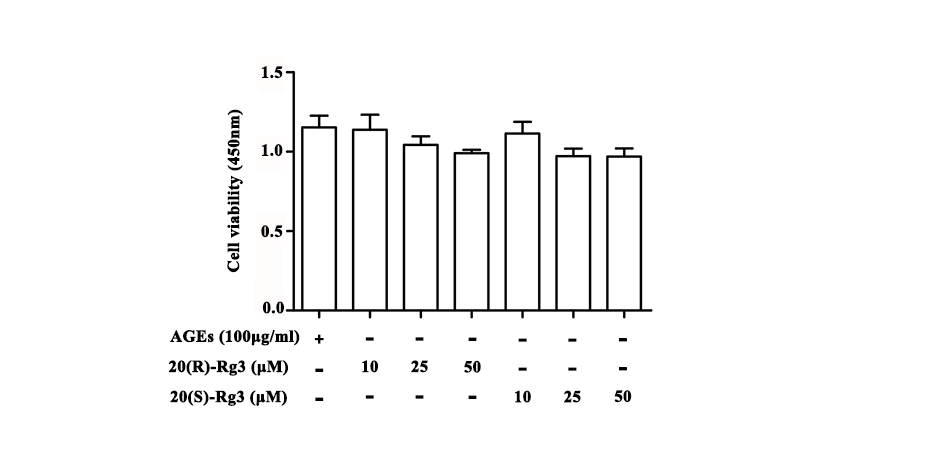

Supplement: Supplementary file 2 [file JCMM-22-3202-s002.tif]

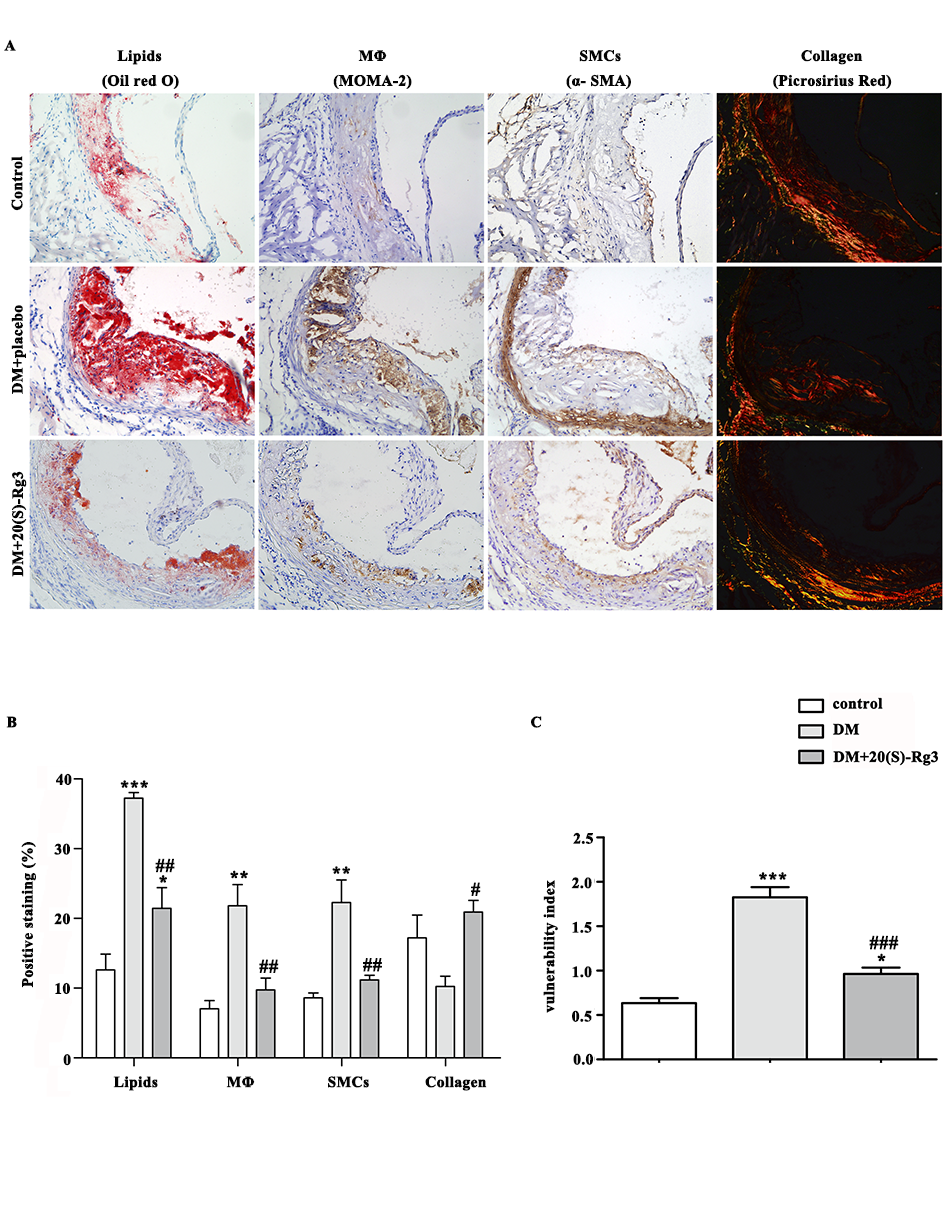

Supplement: Supplementary file 3 [file JCMM-22-3202-s003.tif]
