# Supplementary material for: Ginsenoside Rg3 stereoisomers differentially inhibit vascular smooth muscle cell proliferation and migration in diabetic atherosclerosis
Source: J Cell Mol Med. 2018 Mar 22;22(6):3202–14. doi: 10.1111/jcmm.13601 (PMC5980205; doi:10.1111/jcmm.13601)
Supplement: Supplementary file 4 [file JCMM-22-3202-s004.docx]

| Models | Binding energy estimated by AutoGrid (kcal mol^-1^) | Hydrogen-bond interaction |
| --- | --- | --- |
| S1 | -49.25 | Tyr473, His323, His449, Tyr327, Cys285, Ser342, Glu343 |
| S2 | -41.07 | Tyr473, His323, His449, Tyr327, Cys285, Ser342 |
| S3 (inverted) | -48.37 | Tyr473, His323, His449, Tyr327 |
| R1 | -50.13 | Ser342, Cys285, Glu343, Ser289, His266 |
| R2 | -45.04 | Ser342, Cys285, Glu343, Ser289 |
| R3(inverted) | -44.44 | Tyr473, His323, His449, Tyr327 |
