# Supplementary material for: Ginsenoside Rg3 stereoisomers differentially inhibit vascular smooth muscle cell proliferation and migration in diabetic atherosclerosis
Source: J Cell Mol Med. 2018 Mar 22;22(6):3202–14. doi: 10.1111/jcmm.13601 (PMC5980205; doi:10.1111/jcmm.13601)
Supplement: Supplementary file 5 [file JCMM-22-3202-s005.docx]

| Models | S1 | S2 | S3 | R1 | R2 | R3 | LT160 | LT127 | apo |
| --- | --- | --- | --- | --- | --- | --- | --- | --- | --- |
| Charge-clamp  Distance(Å) | 19.8  ±0.9 | 22.7  ±0.7 | 19.3 ±1.1 | 22.0 ±0.6 | 19.3 ±0.8 | 19.2 ±0.9 | 20.3 ±0.7 | 19.8 ±0.8 | 22.8 ±0.6 |
| ∆G/MMPBSA（kcal.mol^-1^） | -53.9  ±3.9 | -48.8 ±4.3 | -42.2 ±6.6 | -53.7 ±5.6 | -49.7 ±7.8 | -44.1 ±6.1 | -59.3 ±5.1 | -52.6 ±5.1 | - |
