# Supplementary material for: Ginsenoside Rg3 stereoisomers differentially inhibit vascular smooth muscle cell proliferation and migration in diabetic atherosclerosis
Source: J Cell Mol Med. 2018 Mar 22;22(6):3202–14. doi: 10.1111/jcmm.13601 (PMC5980205; doi:10.1111/jcmm.13601)
Supplement: Supplementary file 6 [file JCMM-22-3202-s006.docx]

| Parameters  (mmol/l) | Non-diabetic apoE-/- | Diabetic apoE-/- | | | | |
| --- | --- | --- | --- | --- | --- | --- |
|  |  | placebo | 20(R)-Rg3 | 20(R)-Rg3  +GW9662 | 20(S)-Rg3 | 20(S)-Rg3  +GW9662 |
| Fasting blood glucose | 6.22±0.31 | 24.65±0.82* | 21.83±2.28* | 24.83±1.76* | 20.35±2.06*# | 23.95±3.02* |
| Total cholesterol | 12.18±3.1 | 16.93±2.87* | 17.71±2.03* | 16.93±2.9* | 15.07±2 | 18.75±3.97* |
| Triglyceride | 0.93±0.15 | 1.25±0.26 | 1.09±0.27 | 1.04±0.09 | 1.0±0.34 | 0.95±0.26 |

*p﹤0.05 v­­s. non-diabetic apoE-/- group; # p﹤0.05 vs. placebo-treated diabetic apoE-/- group
